# Differences in Circulating Endothelial Progenitor Cells among Childhood Cancer Survivors Treated with and without Radiation

**DOI:** 10.13188/2380-6842.1000005

**Published:** 2015-08-05

**Authors:** Kamnesh Pradhan, Julie Mund, Jamie Case, Samir Gupta, Ziyue Liu, Wambui Gathirua-Mwangi, Anna McDaniel, Jamie Renbarger, Victoria Champion

**Affiliations:** 1 Department of Pediatric Hematology-Oncology, Riley Hospital for Children, Indianapolis, Indiana, USA; 2 Melvin and Bren Simon Cancer Center, Indiana University Melvin, Indiana, USA; 3 Department of Pediatrics, Scripps Clinic Medical Group, Center for Organ and Cell Transplantation, La Jolla, California, USA; 4 Herman B Wells Center for Pediatric Research, Indiana University School of Medicine, Indianapolis, Indiana, USA; 5 Scripps Clinic Medical Group, Center for Organ and Cell Transplantation, La Jolla, California, USA; 6 Division of Infectious Diseases, Department of Medicine, Indiana University School of Medicine, Indianapolis, Indiana, USA; 7 Division of Biostatistics, Indiana University School of Medicine, Indianapolis, USA; 8 Fairbanks School of Public Health, Indianapolis, Indiana, USA; 9 University of Florida School of Nursing, Gainesville, Florida, USA; 10 Indiana University School of Nursing, Indianapolis, Indiana, USA

**Keywords:** Pediatric cancers, Endothelial cell functions, Long-term toxicities, Radiation induced late effects, Childhood cancer survivors

## Abstract

Radiation during childhood cancer treatment increases the propensity to atherosclerotic cardiovascular disease among adult survivors of childhood cancer. This is thought to be mediated through the damage to the underlying vascular endothelium. Endothelial progenitor cells (EPCs) involved in vascular endothelial repair after its damage may be affected by radiation therapy but have never been investigated in adult survivors of childhood cancer. In this pilot study, utilizing multi-parametric flowcytometry, endothelial colony forming cells (ECFCs), which are the bonafide EPCs, and circulating endothelial cells (CECs), which are not EPCs, were compared between adult survivors of childhood cancer with or without radiation exposure. In addition, their associations with blood-pressure, physical activity and diet were examined. Survivors who received radiotherapy had lower ECFCs and CECs (p<0.05) compared to those without it. Significant positive correlations included physical activity with ECFCs and diet with CECs, while blood-pressure negatively correlated with ECFCs. Further evaluation is needed to examine the effect of radiation and modifiable risk factors on ECFCs and CECs. The preliminary findings from this study suggest evidence of the role of ECFCs as biomarkers of vascular injury following treatment for childhood cancer that may help in early identification of survivors at risk for cardiovascular disease.

## Introduction

Although considerable progress has been made in the treatment of childhood cancers, survivors experience significant late-effects such as atherosclerotic cardiovascular disease at a relatively higher rate compared to non-cancer sibling controls [1-3]. The pathogenesis of atherosclerotic cardiovascular disease is rooted in the vascular endothelial damage brought about by radiation and chemotherapy drugs like anthracyclines and cisplatin. The vascular endothelial damage is accelerated with cardiovascular risk factors such as hypertension or lifestyles such as physical inactivity [4-6]. *In vitro* studies have shown that radiation therapy causes the endothelial progenitor cells of the vascular endothelium, called endothelial colony forming cells (ECFCs), to undergo large-scale senescence, a forerunner of vascular damage and subsequent atherosclerosis [7]. Circulating ECFCs have robust proliferative potential and form perfused new blood vessels *in vivo,* thereby playing an important role in the repair of damaged vascular endothelium [8,9]. Utilizing a novel multi-parametric flow-cytometry (MPFC) protocol, our group has phenotypically defined the ECFCs by the expression of CD31^+^, CD34^bright^, CD45^−^, AC133^−^, CD14^−^, CD41a^−^, CD235a^−^ and LIVE/DEAD Violet^−^ antigens [10,11]. Our group then phenotypically sorted these cells and validated their endothelial characteristics by outgrowth cell culture and expanded endothelial phenotyping, they have colony forming and proliferative potential and formed new blood vessels *in vivo* [11]. Endothelial cells that express CD31, CD45^−^, CD34^dim^ and AC133^−^ antigens are mature, apoptotic cells that cannot form *in vivo* blood vessels, are sloughed off from the vessel wall and are termed circulating endothelial cells (CECs) [10,11]. To verify the apoptotic nature of these cells, our group magnetically isolated these cells and further characterized them through further endothelial cell phenotyping, cell culture where they were unable to form colonies, and apoptotic stains [11].

In contrast to radiation, lifestyle factors such as physical activity has a very favorable effect on endothelial structure and function through its effect on endothelial progenitor cells as seen in the adult non-cancer population [12,13]. Therefore, we decided to examine the differences among ECFCs and CECs in survivors with and without radiation exposure and their association with cardiovascular risk factors such as blood-pressure and lifestyle factors such as physical activity and diet.

## Materials and Methods

A pilot study was planned to recruit 2 cohorts consisting of young adult survivors (current age ≥18 years but < 30 years) who had received chemotherapy but differed by their exposure to radiotherapy. The survivors were recruited over a 12 month period from a large, university-based, tertiary children’s outpatient oncology clinic located in the Midwestern United States. Study protocol was approved by the institutional review board.

Self-reported physical activity and diet were obtained from a questionnaire adapted from the Behavioral Risk Factor Surveillance System (BRFSS) survey questions [14,15]. A higher score implied higher self-reported physical activity and a diet higher in fruits and vegetables.

### Mononuclear cell isolation, flow cytometry acquisition and analysis

**Figure 1:**
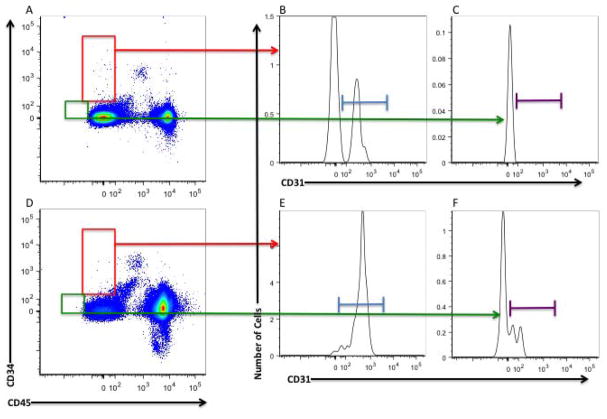
Representative gating strategy of ECFC and CEC populations using Flow Cytometry. Following exclusion of dead cells, low side scatter mononuclear cells were plotted to show CD34 and CD45 (Radiation (XRT): A, Non-XRT: D). CD45^−^CD34^+^ cells were gated [red] and subsequently gated for CD31 to verify endothelial phenotype (XRT: B; Non-XRT: E). CD45^−^CD34^dim^ cells were gated [green] and subsequently gated for CD31 to verify endothelial phenotype (XRT: C; Non-XRT: F).

A 7-color MPFC assay was conducted based on previously published studies by our group [16,17]. Briefly, mononuclear cells isolated with ficoll density gradient centrifugation were stained with antibodies against cell surface antigens, CD34, AC133, CD31, CD45, CD14, and CD16 as well as a viability marker (LIVE/DEAD) and glycophorin A, for the exclusion of dead cells and red blood cells respectively. Fluorescence minus one (FMO) controls were used as positive gating controls are shown in Figure 1. The frequency of phenotypically defined cell populations were acquired using an LSR II flow cytometer equipped with 405 nm violet, 488 nm blue, and 633 nm red laser. All samples were run uncompensated and analyzed using FlowJo 9.7.4 software (Tree Star Inc.) as previously described. The CECs and ECFCs were expressed as the percentage of the total mononuclear cell population.

### Statistical analyses

Categorical variables were compared between groups using the Fisher’s exact test. For comparing continuous variables, Student’s t-test was used and for correlations among continuous variables, Pearson’s correlations were tested unless normality assumptions were violated, in which case Wilcoxon’s rank sum tests were used for comparisons and Spearman’s rank based for correlations. All analyses were performed using the SAS version 9.3 (SAS Institute, Cary, North Carolina).

## Results

**Figure 2:**
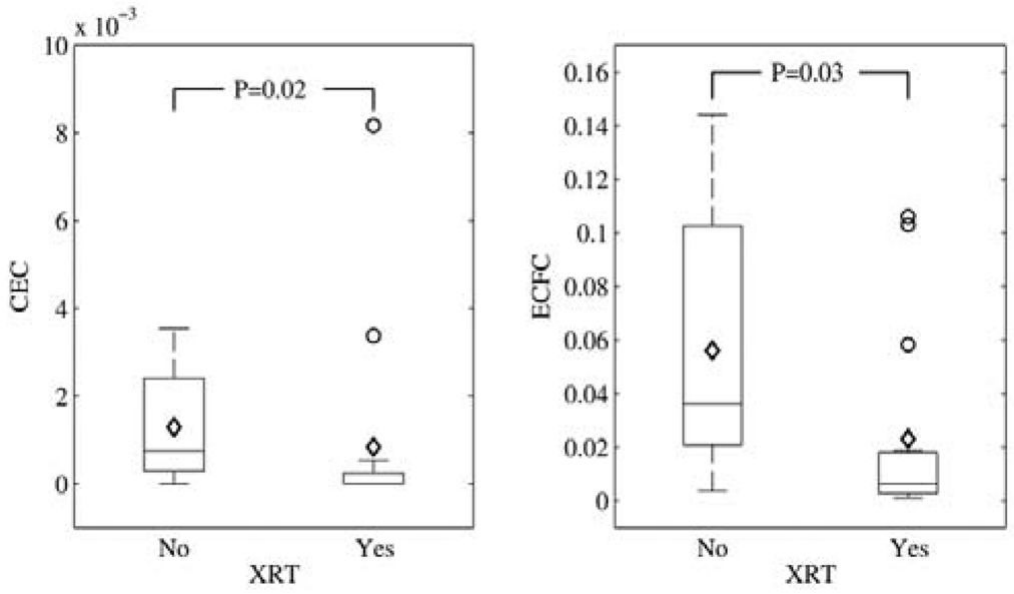
Box-plots showing levels of circulating endothelial cells (CECs) and endothelial colony forming cells (ECFCs) among childhood cancer survivors treated with and without Radiation (XRT).

Twenty-four childhood cancer survivors were recruited over 12 months but were not evenly distributed based on radiation exposure. There were no differences between the 2 groups based on age and gender. Of the 24 survivors, 15 received chemotherapy with radiation. Detailed radiation fields and doses are shown in Table 1. Eight survivors with acute leukemia received the same chemotherapy as those with radiation exposure but without any radiotherapy. Majority of the survivors (n=17) had a history of acute leukemia while the rest had solid tumors (6 with brain tumors and 1 with Ewing’s sarcoma). The average years from end of treatment to study visit was 8.5 years (SD=3). Eighteen out of 24 survivors received anthracyclines, of which 9 received anthracyclines with radiation. Six of the 24 survivors received cisplatin with radiation without any anthracyclines. The mean percentages of ECFCs for the radiated cohort was 0.0231 (SD=0.036) and for the non-radiated cohort was 0.0561 (SD=0.048). The mean percentages of CECs for the radiated cohort was 0.000837 (SD=0.0022) and for the non-radiated cohort was 0.00129 (SD=0.0012). Those who received radiation had significantly lower levels of ECFCs and CECs (p=0.03 and 0.02 respectively) compared to those who received chemotherapy without radiation (as shown in Figure 2). Among all survivors, there was a significant negative correlation between systolic blood pressure and ECFCs (p=0.015, spearman rho=0.48). In addition, among all survivors, there was a significant positive correlation between self-reported physical activity and ECFCs (p=0.0339, spearman rho=0.43) and diet and CECs (p=0.02, spearman rho=0.45).

## Discussion

This is the first study showing an inhibitory effect of radiotherapy on both the mature (CECs) and progenitor endothelial cell (ECFC) populations among survivors late into the survivorship period (as shown in Table 2). This significant decline of both endothelial cell populations in the radiated cohort confirms the pre-clinical studies showing sensitivity of the ECFCs to radiation greater than 10 GY leading to large-scale radiation induced senescence a cellular phenotype linked to the premature development of atherosclerosis and vasculopathies [17,18]. However, the pre-clinical studies were carried out immediately after radiation therapy in contrast to our survivor population where a significant decline among endothelial cells was seen many years following it (median: 9.6±3 yrs). This long-term effect may indicate lesser capacity for the ECFCs to repair, remodel, and form new perfused blood vessels. The persistent vessel wall damage and a lower capacity to form collateral vessels accelerate atherosclerosis. This may also explain the significant decline in CECs, which are mature, apoptotic cells that are typically sloughed off from the vessels during vessel remodeling.

The significant negative association of systolic blood pressure with ECFCs greatly underscores the importance of altering modifiable risk factors such as hypertension that can adversely affect vascular health and predispose to atherosclerotic cardiovascular disease [19]. The strong positive correlation of physical activity with ECFCs should be encouraging to survivors and is consistent with recent findings where physical activity enhanced endothelial progenitor cell and endothelial function in the non-cancer population [12]. The ECFCs may mediate the role of physical activity in maintaining robust cardiovascular health. Therefore, physical activity among cancer survivors could potentially alleviate the detrimental effects of radiation on the vascular endothelium. The reason for the significant association between CECs and a diet higher in fruits and vegetables as seen in our study is not known and is being further investigated at this time.

The limitations of this study are the small sample size, cross-sectional study design and use of self-report to assess physical activity and diet measures. However, despite these limitations we found statistically significant relationships underscoring the probability that our findings can be duplicated. Although we had information of the fields and doses of radiation, being a pilot study, we did not have the ability to look at the relationship between these radiation parameters and ECFCs and CECs. In addition, contemporary pediatric cancer therapies are complex involving a combination of multiple different chemotherapy regimens with or without radiation. Evaluating and comparing chemotherapy regimens among the 2 cohorts and then adjusting for the different treatment effects was beyond the scope of this pilot study. So we examined differences in regard to radiation exposure that is the strongest causation of vascular dysfunction. A larger study adjusting for other treatment effects is warranted.

In conclusion, the novel findings from this pilot study show the negative effect of radiation exposure on endothelial progenitor cells such as ECFCs. The role of the ECFCs as a potential biomarker to identify childhood cancer survivors at risk for atherosclerotic cardiovascular disease and the effect of lifestyle changes such as physical activity on this cellular population will need to be confirmed in a larger, longitudinal study.

## Figures and Tables

**Table 1:** Treatment groups of all survivors.

Treatment Groups	Acute Leukemia (n=17)	Solid Tumors (n=7)	Total
With XRT	8	7	15
Without XRT	9	--	9
Cisplatin	--	--	0
Anthracyclines	9	0	9
Cisplatin + XRT	--	6	6
Anthracyclines + XRT	8	1	9
SCT	3	--	3

XRT: Radiation Therapy; SCT: Stem Cell Transplantation

**Table 2:** Clinical data of the survivors who received Radiotherapy.

Primary diagnosis	Field of XRT	XRT dose (Gy)	Time-Interval between XRT and Study Visit (years)
B-ALL with testicular relapse	Bilateral Testicles	21	8
B-ALL with CNS+	Cranial	18	16
B-ALL relapse	CSI + Cranial boost	18 + 6	10
APML with CNS+	CSI	21.6	5
B-ALL with relapse	CSI + Cranial boost + TBI	18 + 6 + 13.5	10
Ph+ ALL	TBI + Cranial boost	12 + 6	7
B-ALL with CNS+	Cranial	18	10
B-ALL with CNS+	CSI	18 + 6	13
Medulloblastoma	CSI + PF boost	23.4 + 55.4	8
Medulloblastoma	CSI + PF boost	23.4 + 55.4	9
CNS/PNET	CSI + tumor bed boost	36 + 50.4	6
Medulloblastoma	CSI + PF boost	36 + 50.5	7
Pineoblastoma	CSI + tumor bed boost	36 + 50.4	7
Medulloblastoma	CSI + PF boost	23.4 + 55.4	13
Ewing’s Sarcoma	Focal (Left Parietal Skull)	45-54	16

XRT: Radiation Therapy; B-ALL: Precursor-B Acute Lymphoblastic Leukemia; CNS+: Central Nervous System is positive for leukemic infiltration; APML: Acute Promyelocytic Leukemia; Ph+ ALL: Philadelphia chromosome ALL; CNS/PNET: Peripheral Neuro-ectodermal Tumor of the Brain; CSI: Cranio-Spinal; boost: Additional XRT; PF: Posterior fossa of the Brain; TBI: Total Body Irradiation; Focal: tumor and surrounding margin only; Gy: Gray (unit of radiation dose)
